# Absence of ERRα in Female Mice Confers Resistance to Bone Loss Induced by Age or Estrogen-Deficiency

**DOI:** 10.1371/journal.pone.0007942

**Published:** 2009-11-20

**Authors:** Catherine Teyssier, Marlène Gallet, Bénédicte Rabier, Laurent Monfoulet, Julien Dine, Claire Macari, Julie Espallergues, Béatrice Horard, Vincent Giguère, Martine Cohen-Solal, Olivier Chassande, Jean-Marc Vanacker

**Affiliations:** 1 Institut de Génomique Fonctionnelle de Lyon, Université de Lyon, Université Lyon 1, Centre National de la Recherche Scientifique, Institut National de la Recherche Agronomique, Ecole Normale Supérieure de Lyon, Lyon, France; 2 Institut National de la Santé et de la Recherche Médicale U 577, Université Victor Segalen Bordeaux II, Bordeaux, France; 3 Institut National de la Santé et de la Recherche Médicale U710, Université de Montpellier II, Montpellier, France; 4 Laboratoire de Biologie Moléculaire de la Cellule, CNRS UMR5239, Ecole Normale Supérieure de Lyon, Villeurbanne, France; 5 The Rosalind and Morris Goodman Cancer Centre, Montréal, Canada; 6 Institut National de la Santé et de la Recherche Médicale U606, Hôpital Lariboisière, Paris, France; Ohio State University, United States of America

## Abstract

**Background:**

ERRα is an orphan member of the nuclear hormone receptor superfamily, which acts as a transcription factor and is involved in various metabolic processes. ERRα is also highly expressed in ossification zones during mouse development as well as in human bones and cell lines. Previous data have shown that this receptor up-modulates the expression of osteopontin, which acts as an inhibitor of bone mineralization and whose absence results in resistance to ovariectomy-induced bone loss. Altogether this suggests that ERRα may negatively regulate bone mass and could impact on bone fragility that occurs in the absence of estrogens.

**Methods/Principal Findings:**

In this report, we have determined the *in vivo* effect of ERRα on bone, using knock-out mice. Relative to wild type animals, female ERRαKO bones do not age and are resistant to bone loss induced by estrogen-withdrawal. Strikingly male ERRαKO mice are indistinguishable from their wild type counterparts, both at the unchallenged or gonadectomized state. Using primary cell cultures originating from ERRαKO bone marrow, we also show that ERRα acts as an inhibitor of osteoblast differentiation.

**Conclusion/Significance:**

Down-regulating ERRα could thus be beneficial against osteoporosis.

## Introduction

Bone is a highly dynamic tissue subjected to active remodeling, an equilibrium between construction by osteoblasts and resorption by osteoclasts. Osteoblast are derived from mesenchymal stem cells (MSCs) and their differentiation is promoted by various factors such as the transcription factors Osterix and Runx2 [1]–[3]. Mature osteoblasts express receptor activator of nuclear factor-κB ligand (RANKL), a protein that will signal through RANK, a member of the tumor necrosis factor receptor superfamily present at the surface of pre-osteoclasts, and induce differentiation of these cells [4]–[7]. On another hand, osteoblasts, as well as other cell types, also express and secrete osteoprotegerin (opg), a decoy receptor that traps RANKL in the extracellular milieu, preventing it from acting on pre-osteoclasts, and thus inhibiting the differentiation of osteoclasts [8], [9].

Stability of bone remodeling also requires other diffusible factors among which estrogens are instrumental. This is particularly illustrated after menopause in aging women, when the fall of circulating level of estrogens leads to enhanced bone remodeling with an excessive resorption [10], [11]. The resulting osteoporosis syndrome is associated with an enhanced fracture risk. Various means are currently available to prevent and treat osteoporosis, most of which mainly aim at inhibiting the excess of bone resorption. These include for example the use of estrogens (natural or synthetic) or of bisphosphonate to reduce osteoclast differentiation or induce their apoptosis, respectively [11].

The effects of estrogens are mediated by two estrogen receptors (ERs) α and β, which act as ligand-dependent transcription factors and belong to the nuclear receptor superfamily [12]. This family also comprises so-called orphan receptors, *i.e.* for which no ligand has been identified to date [13]. Estrogen-Receptor Related receptor α (ERRα) was among the first orphan receptors isolated, based on its sequence similarity to ERα [14], [15]. Despite this close proximity, ERRα does not bind estrogen nor any identified natural ligand [16]. ERRα is expressed in several tissues both during embryonic development and in the adult [17]. A number of studies have identified ERRα as a major actor in the regulation of energy metabolism in oxidative tissues such as heart or slow-twitch skeletal muscle [18, 19 for reviews]. This receptor is indeed involved in the regulation of energy uptake, storage and consumption as well as in mitochondrial biogenesis and function and cardiac response to stress. Most of these effects are thought to depend on the PGC-1α coactivator, with which ERRα physically interacts. High expression of ERRα in various human tumors (originating from such organ as breast, colon and ovary) correlates with a poor prognosis [reviewed in 20]. Proliferation of various cancer cell lines can be inhibited by a synthesis modulator of ERRα, but not by its mere siRNA-mediated knocking-down [21]. ERRα is highly expressed in all ossification zones during mouse development, as well as in osteoblastic lines and normal human bones [22]. Furthermore, a polymorphic variant of the human ERRα promoter has been associated with variable bone mineral density (BMD) in premenopausal women [23]. Although the molecular functions of ERRα in bone cells have not been determined, osteopontin (opn), an inhibitor of bone mineralization [24]–[30], has been identified as a positive ERRα transcriptional target [31]–[33].

Here, we show that ERRαKO female mice resist to age-induced bone loss and present a more elevated bone formation rate as compared to wild type animals. Remarkably, ERRα deficiency also conferred resistance to bone loss induced by estrogen withdrawal. Noteworthy, no phenotype was observed in the bones of ERRα-deficient males. ERRαKO originating MSCs displayed enhanced capacities to differentiate into osteoblasts, together with a lower opn expression, as compared to wt MSCs. Altogether our results reveal the role of ERRα in the control of bone density and suggest important cross talks between ERRα and hormonal signalling pathways in bone physiology.

## Results and Discussion

### ERRα Deficiency Affects Bone Aging in Female, but Not in Male, Mice

**Figure 1:**
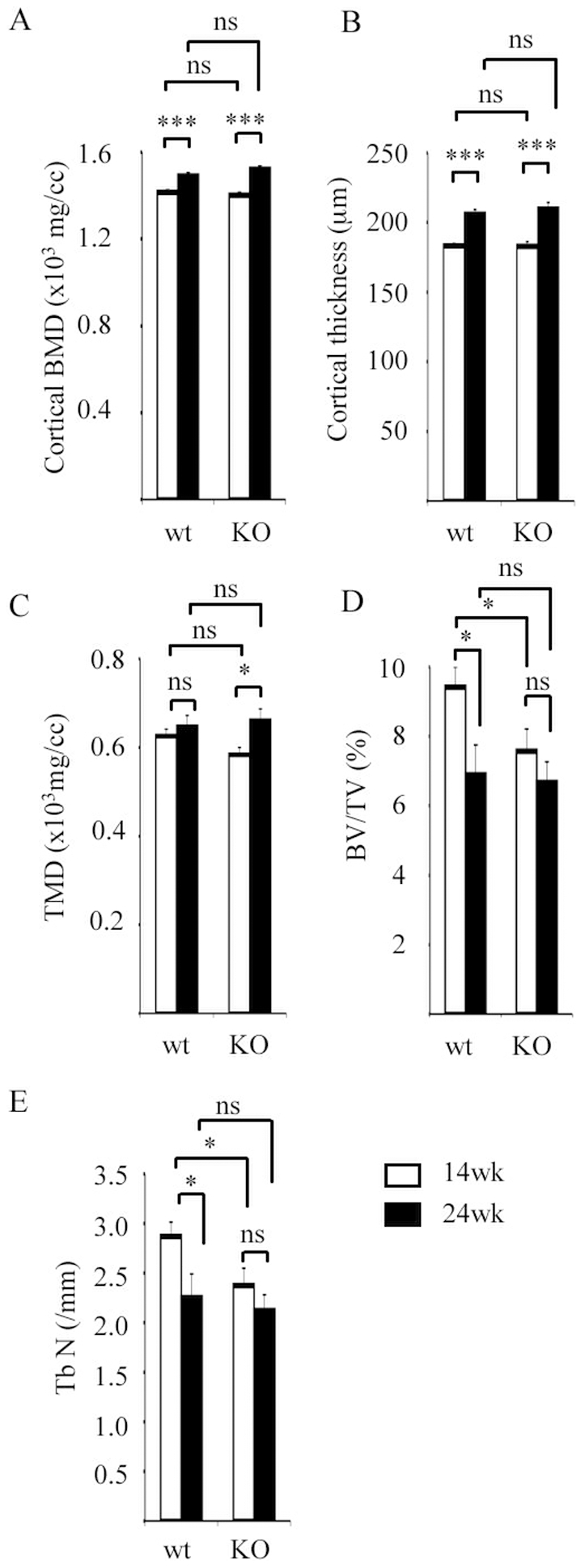
Bone phenotype of ERRαKO females mice. Cortical bone mineral density (BMD; **A**), cortical thickness (**B**), trabecular mineral density (TMD; **C**), bone volume fraction (BV/TV; **D**) and trabecular number (TbN; **E**) were determined at 14 (white bars) and 24 wk (black bars) in the femur of female wt and ERRαKO mice (n = 6 to 9). Errors bars represent s.e.m. ns: not significant; *:p<0.05; *** p<0.005.

The structural parameters of cortical and trabecular bone were measured in the femur diaphysis and metaphysis, respectively, of wild type (wt) and ERRα knockout female mice at 14 and 24 weeks. Cortical bone mineral density (BMD) and thickness augmented with age in a similar manner in both genotypes (Fig. 1A and B). The trabecular mineral density (TMD) was slightly more elevated (but not statistically significant) in wt animals as compared to ERRαKO, but significantly increased with time in the latter genotype (Fig. 1C). Lower trabecular bone volume (BV/TV; Fig. 1D) and trabecular number (TbN; Fig. 1E) were observed in ERRαKO animals relative to wt ones at 14 wk. Interestingly whereas these two parameters were significantly reduced at 24 wk in wt animals as an effect of age, no significant variation occurred in ERRαKO mice. These results indicate that ERRαKO female mice, although deficient in bone trabeculae at maturity (14 wk), are resistant to age-related bone loss. Strikingly no difference was observed in any of the structural parameters of both cortical and trabecular bone between wild type and mutant male mice (Table 1), indicating a gender-dependent effect of the absence of ERRα.

**Table 1:** Trabecular bone parameters of wt and ERRαKO male mice (n = 8 to 10).

	14 wk		24 wk		Orx^1^	
	wt	ERRαKO	wt	ERRαKO	wt	ERRαKO
TMD^2^ (mg/cc)	**752** +/− 43	**770** +/− 33	**731** +/− 8	**694** +/− 7	**598** +/− 14	**570** +/− 19
BV/TV^3^ (%)	**19.3** +/− 1.9	**19** +/− 0.7	**9.4** +/− 0.8	**10** +/− 0.4	**5.9** +/− 0.2	**5.5** +/− 0.5
TbN^4^ (/mm)	**7.7** +/− 0.6	**7.5** +/− 0.4	**4.7** +/− 0.3	**4.5** +/− 0.5	**3.6** +/− 0.3	**3.9** +/− 0.3

1 : orchidectomized animals.

2 : trabecular mineral density.

3 : bone volume/tissue volume.

4 : trabecular number.

**Figure 2:**
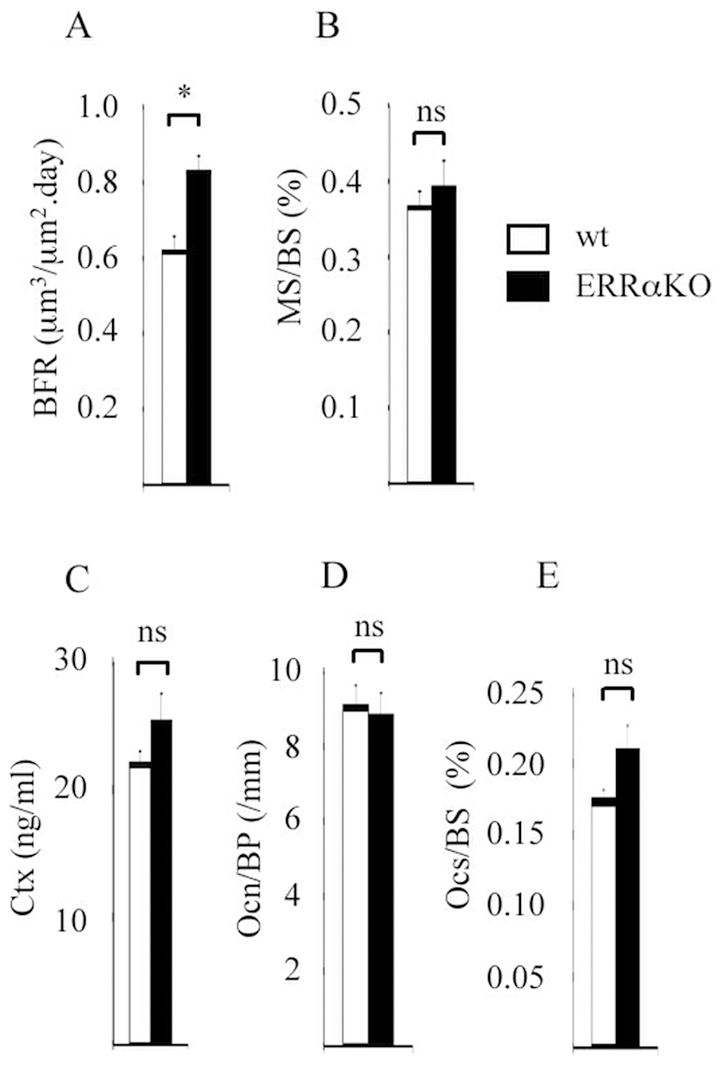
Dynamic parameters of ERRαKO female mice. Bone formation rate (BFR; **A**) and mineralizing surface per trabecular bone surface (MS/BS; **B**) were measured in the metaphysis of 14 wk old mice. **C**) Concentration of the C-terminal fragments of collagen 1 (Ctx) were measured in the serum of 14 wk old females. Osteoclast number per bone perimeter (OCn/Bp; **D**) and osteoclast surface per bone surface (OCs/Bs; **E**). n = 8. White bars: wild type; black bars: ERRαKO mice. Error bars represent s.e.m. ns: not significant; *:p<0.05.

The evolution of bone between wild type and ERRαKO females prompted us to examine the parameters of bone resorption and bone formation at 14 wk of age (*i.e.* before a significant difference could be observed between genotypes). Bone formation rate, which represent the activity of osteoblasts, was more elevated in ERRαKO females than in wt animals (Fig. 2A). The surface occupied by osteoblasts was identical in both genotypes (Fig. 2B). Serum markers of bone resorption (C-terminal fragment of collagen 1 [Ctx], indicative of osteoclast activity) were not different between wild type and mutant females, indicating that bone resorption was not affected by the ERRα deficiency (Fig. 2C). Consistently, a comparable number of osteoclasts (Fig. 2D), spaning an identical relative surface (Fig. 2E) were detected in both strains.

Altogether this suggests that the resistance of ERRαKO animals to age-induced bone loss could be due to an enhanced osteoblastic activity, and not to variation in osteoclast-related parameters.

### ERRα Negatively Regulates Osteoblast Differentiation

**Figure 3:**
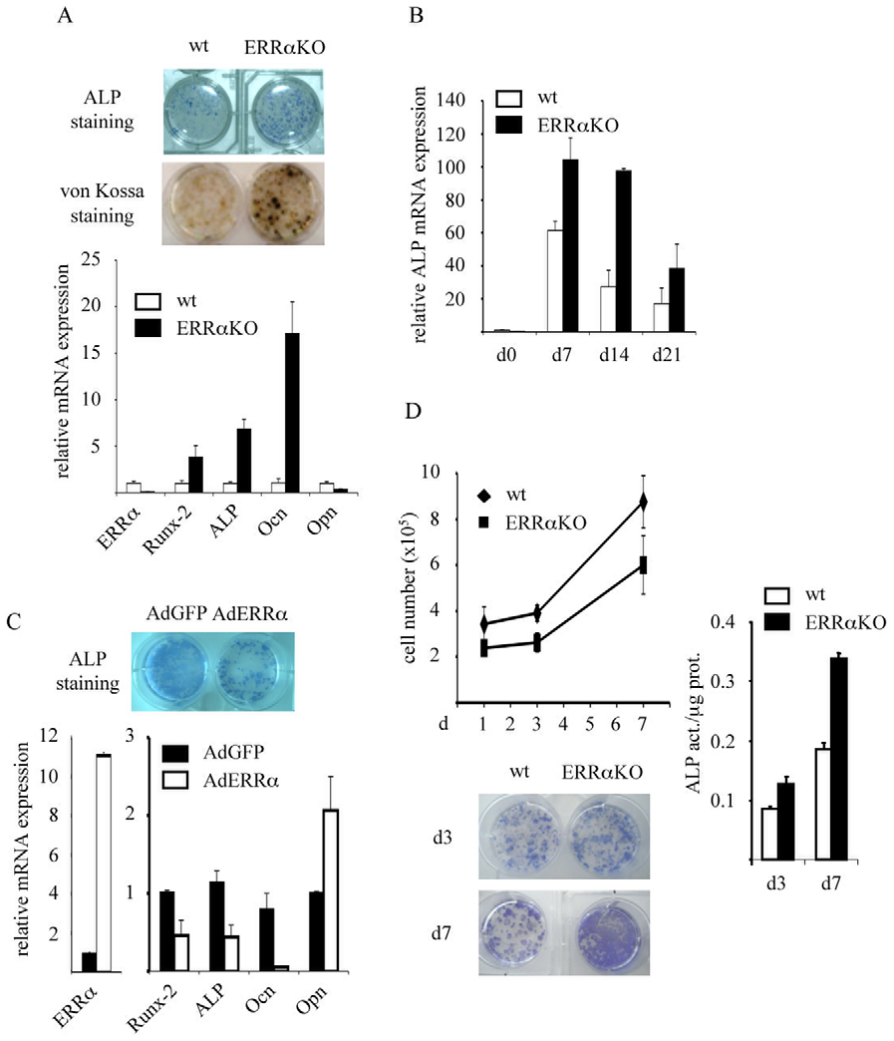
*Ex vivo* differentiation of bone marrow osteoblast precursors originating from ERRαKO mice. **A**) Upper panels: Alkaline phosphatase (ALP)- and von Kossa staining after 7 d and 21 d, respectively, in differentiation medium. Lower panel: expression of osteoblast differentiation markers measured by QPCR after 7 d in differentiation medium. Data are expressed relative to wild type level. Ocn: osteocalcin; opn: osteopontin. **B**) Time course of ALP expression after switch to differentation medium in wild type- (white bars) and ERRαKO- (black bars) originating cells measured by QPCR. Data are expressed relative to wild type level at d0. **C**) Rescue of the differentiation phenotype by infection with an ERRα-encoding *vs* a GFP-encoding adenovirus. Upper panel: ALP staining; lower panel: expression of osteoblast differentiation markers determined by QPCR. Adenoviruses were added together with the differentiation medium and the experiment was stopped after 7 d. Data are expressed relative to AdGFP infected cells. **D**) Proliferation of osteoblast precursors. Upper left panel: time course of cell number; lower left panel: time course of ALP staining; right panel: time course of ALP enzymatic activity relative to protein content. Time is indicated after switch to differentiation medium. All experiments were performed at least three times using female bone marrow. Staining views represent a single typical experiment; expression data and cell counting represent a typical experiment performed in triplicate with error bars indicating S.D.

We thus analyzed the capacities of ERRαKO bone marrow mesenchymal cells to differentiate into osteoblasts *ex vivo*. After seven days in the appropriate medium, ERRα mutant cells produced a greater number of differentiation foci as estimated by alkaline phosphatase (ALP) staining (Fig. 3A). This was accompanied by a higher mineralization activity, as revealed by von Kossa staining. As measured by quantitative PCR, the expression of various osteoblast differentiation markers, such as Runx-2, ALP or osteocalcin was found more elevated in ERRαKO cells by quantitative PCR. In contrast, opn, an ERRα target gene [31]–[33], was less expressed in ERRαKO cells than in wild type cells. We also analyzed the kinetics of ALP expression during osteoblast differentiation (Fig. 3B). In ERRαKO cells, ALP expression followed the same profile as in wild-type cells but was more elevated at each time point. The phenotype displayed by mutant cells was rescued by infection with an ERRα-encoding adenovirus (AdERRα; Fig. 3C). Indeed, ALP staining was reduced by AdERRα infection as compared to cells infected with a GFP-encoding adenovirus. AdERRα also reduced the mRNA expression of Runx-2, ALP and osteocalcin. This repression was not a general, non-specific phenomenon since osteopontin expression was enhanced by AdERRα, as expected. This suggests that the capacities of MSCs to differentiate into osteoblasts are enhanced in the absence of ERRα and that the number of precursor cells is not modified. The results above may reflect an effect of the absence of ERRα on osteoblast proliferation rather than on differentiation. To evaluate this hypothesis, we determined the growth capacities of bone marrow cells (Fig. 3D). ERRαKO cells displayed identical growth rate as wild-type cells, as estimated by determining cell number after 3 and 7 days in culture. However, under these conditions, ERRαKO cells still displayed a higher ALP expression as measured by staining and mRNA expression. We thus concluded that the absence of ERRα enhanced the osteoblastic differentiation of bone marrow cells without modifying their proliferation capacities. This is in agreement with recent results showing that the inhibition of ERRα expression in mammary cells does not modify proliferation [21]. Our results are in contrast with previous data suggesting that ERRα promotes osteoblast differentiation [34]. However, it is worth noting that our study analyzed femoral mesenchymal cells (which form endochondral bone, *i.e.* requiring a cartilage anlagen) derived from ERRαKO mice, whereas Bonnelye et al. [34] used transiently transfected rat calvarial cells (which form intramembranous bone). These methodological differences might account for the apparent discrepancies found between both studies. In contrast, our data are in agreement with published results in which MSCs originating from an independent ERRαKO strain were found more prone to differentiate into osteoblasts [35]. Interestingly this report also show that ERRα concomitantly promotes adipocyte differentiation of MSCs, suggesting that the receptor modulates an inverse relationship between adipogenic and osteogenic commitment of MSCs. In further support of our data, it is also worth noting that the closely related ERRγ receptor has recently been found to inhibit BMP2-induced osteoblast differentiation *in vitro* as well as BMP2-induced ectopic bone formation *in vivo* [36]. Elevated osteoblast differentiation *ex vivo* in the absence of ERRα is furthermore consistent with the more elevated BFR found *in vivo* in ERRαKO mice. Furthermore, opn has been shown to inhibit bone mineralization [24]–[30]. A lower level of opn expression in the absence of ERRα could thus result in higher mineralization as observed in aging ERRαKO mice.

### ERRα Deficiency Protects Females from Bone Loss Resulting from Sex Hormone Ablation

**Figure 4:**
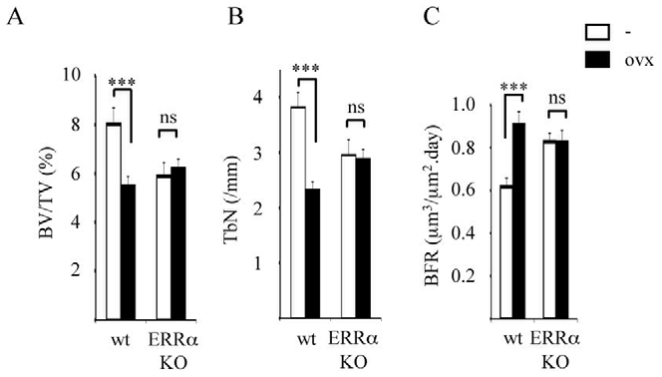
Bone structural parameters of ovariectomized ERRαKO mice. Bone volume fraction (BV/TV; **A**), trabecular number (TbN; **B**), and bone formation rate (BFR; **C**) were determined in the femur 4 wk after surgery (performed at 10 wk of age) in sham (white bars) or ovariectomized (black bars) wild type and ERRαKO mice. n: 8. Errors bars represent s.e.m. ns: not significant; *:p<0.05; *** p<0.005.

Normal aging causes a moderate bone loss as the result of a progressive imbalance between bone formation and bone resorption. This equilibrium is also disrupted in favor of bone resorption upon sex hormone deficiency which leads to a severe bone loss, as can be observed in women after menopause or in mice after ovariectomy. Since bone formation is more elevated in ERRαKO we hypothesized that this phenomenon might compensate for the remodeling imbalance induced by sex-hormone withdrawal. Gonadectomized wild type females showed an important decrease of relative bone volume (Fig. 4A) and trabecular number (Fig. 4B) four wks after surgery. Strikingly, none of these parameters were significantly affected in ovariectomized ERRαKO animals, indicating that cancellous bone was preserved in mutant mice. Bone formation rate was enhanced in wt females as marker of enhanced bone remodeling (Fig. 4C). In contrast, this parameter did not vary in ERRαKO mice according to the hormonal status. In males, orchidectomy resulted in similar variations in mineral density, bone volume and trabecular number in wild type and mutant mice (Table 1), indicating suggesting that bone was equally affected, independently of the genotype. We thus concluded that the absence of ERRα completely protects against the bone loss induced by deficiency in sex hormone action in females, but not in males.

The mechanisms responsible for this phenomenon are not identified but it is tempting to speculate that *i*) the higher differentiation capacities of MSCs in osteoblasts in the absence of ERRα as well as *ii*) the low expression of opn in ERRαKO osteoblasts could be involved in sparing bone under estrogen deficiency challenge. Indeed, it has been shown that opn-deficient mice are resistant to ovariectomy-induced bone loss [37].

In summary, our data show that the absence of ERRα leads to a resistance in age- as well as estrogen-deficiency-dependent bone loss exclusively in female mice. This finding may have important consequences for the treatment of osteoporosis following menopause, and will need careful investigation to unravel the mechanisms through which ERRα acts in osteoblast differentiation and bone mineralization *in vivo*.

## Materials and Methods

### Animals

ERRαKO animals have been described elsewhere [38]. All animal experiments were performed in the Plateau de Biologie Experimentale de la Souris (PBES; ENS Lyon) under Animal care procedures, conducted in accordance with the guidelines set by the European Community Council Directives (86/609/EEC) and approved by the local ethical committee. Animals were in C57black6 background and, except were stated, had access to food and water *ad libitum*. Mice were sacrificed by cervical dislocation at 10 a.m.

For surgery, animals were anesthetized with sodium pentobarbital. Testes were ligatured and cut through an incision in the scrotum. Ovaries were removed through an incision in the flanks. Animals were sacrificied 4 wk after operation. Statistical significance was analyzed using one-way ANOVA.

### X-ray Micro-Computed Tomography Analysis

3D microarchitecture of the femur was evaluated using a high-resolution (8 µm) microtomographic imaging system (eXplore Locus, GE, USA). A 3D region within the secondary spongiosa in the proximal metaphysis of the femur was reconstructed, beginning 500 µm proximal to the growth plate and extending to 1.5 mm. Cortical bone was reconstructed from a 1 mm thick region of interest centered on the diaphysis, 5 mm distal from the proximal growth plate. Morphometric parameters were computed using the Advanced Bone Analysis Microview Software (GE).

### Histomorphometric Analysis

For histological analyses, undecalcified bones were fixed, deshydrated in ethanol solutions at 4°C and embedded in methyl-methacrylate according to standard protocols. For each femur, 7 µm thick longitudinal sections, parallel to the sagittal plane, were prepared. Bone sections were stained with von Kossa/van Giseon reagent, images were captured using a Nikon eclipse 80i microscope and analyzed using NIS Elements AR 2.30 software (Nikon). To determine bone formation parameters, calcein (10 mg/kg body weight) was injected to mice i.p. 5 and 2 d before sacrifice. Bone formation rate was calculated as interlabeled widths/interval time. To determine bone resorption parameters, sections were stained for tartrate-resistant acid phosphatase (TRAP). TRAP-positive multinucleated cells attached to bone were scored as osteoclasts and results were expressed as number of osteoclasts per trabecular bone perimeter. Bone-related degradation products from Collagen 1 (Ctx) in serum were measured by immunosorbent assay, using a RatLaps ELISA kit from Nordic Bioscience Diagnostics A/S (Herlev, Denmark), following the recommendations of the manufacturers. Measurements were performed after overnight fasting.

### Bone Marrow Cultures

Primary MSCs were collected from bone marrow of femur and tibia of 12-week old mice and cultured in six-well dishes (4×10^6^ cells/well) in MEMα containing 10% fetal calf serum with 100 U/ml penicillin G and 100 µg/ml streptomycin. For osteoblastic differentiation, after 5 d of culture the medium was supplemented with 50 µg/ml L-ascorbic acid and 10 mM β-glycerophosphate and replaced every 2 to 3 days for 3, 7, 14 or 21 days. Osteoblastic differentiation was evaluated by ALP staining and ALP enzymatic activity. For ALP staining, cells were rinsed twice with PBS, and then fixed with 4% (v/v) formaldehyde for 5 min. After 3 washes with H_2_O, cells were incubated 30 min with Fast Blue RR salt and Naphtol AS-MX Phosphate Alkaline solution 0,25% (Sigma Aldrich) in the dark. For ALP enzymatic activity measurement, cells were lysed with Lysis Buffer (50 mM Tris, HCl pH 7,5; 0,1% Triton-X100; 0,9% NaCl). Cell lysates were incubated with 0,2 M of Naphtol Alkaline solution (Sigma Aldrich) and the conversion of *p*-nitrophenyl phosphate to *p*-nitrophenol was spectrophotometrically determined and normalized to cellular protein content by using detergent compatible Bradford reagent (Pierce). For proliferation tests, viable cells were visually counted after resuspension and trypan blue coloration. Adenoviral vectors expressing GFP or ERRα have been previously described [39] and kindly donated by Anastasia Kralli.

### Expression Analysis

RNAs were purified using Guanidinium thiocyanate/phenol/chloroforme extraction. Total RNAs were reverse transcribed using Superscript II retrotranscription kit (Invitrogen). Quantitative PCR were performed using the SYBR GREEN Jump Start kit (Sigma Aldrich) in duplicate on an ABI apparatus using standard procedure. Expression data were normalized to the expression of the 36b4 housekeeping gene. Sequence of the primers used in this study:

36b4: 5′-ACCTCCTTCTTCCAGGCTTT-3′ and 5′-CCCACCTTGTCTCCAGTCTTT-3′; ALP: 5′-GCCCTCCAGATCCTGACCAA-3′ and 5′- GCAGAGCCTGCTGGTCCTTA-3′; ERRα: 5′-CAAACGCCTCTGCCTGGTCT-3′ and 5′-ACTCGATGCTCCCCTGGATG-3′; OCN: 5′-ACCTCACAGATGCCAAGCCC-3′ and 5′-AGCGCCGGAGTCTGTTCACT-3′; OPN: 5′-TCTCCTTGCGCCACAGAATG-3′ and 5′-TCGTCCATGTGGTCATGGCT-3′; Runx-2: 5′-GACGTGCCCAGGCGTATTTC-3′ and 5′-GGAACTGCCTGGGGTCTGAA-3′.
